# A Prospective Study on Child Abuse and Elder Mistreatment: Assessing Direct Effects and Associations With Depression and Substance Use Problems During Adolescence and Middle Adulthood

**DOI:** 10.1093/geroni/igab028

**Published:** 2021-08-20

**Authors:** Todd I Herrenkohl, Karen A Roberto, Lisa Fedina, Sunghyun Hong, Jasmine Love

**Affiliations:** 1 School of Social Work, University of Michigan, Ann Arbor, Michigan, USA; 2 Center for Gerontology & Institute for Society, Culture and Environment, Virginia Polytechnic Institute and State University, Blacksburg, Virginia, USA

**Keywords:** Alcohol use and abuse, Caregiving by adult children, Child maltreatment, Family violence

## Abstract

**Background and Objectives:**

We examined the prospective association between 2 measures of child abuse, one based on official child welfare records and the other based on parent self-reports, and the perpetration of elder mistreatment by an adult child. We also examined measures of adolescent and adult depression and substance use problems as predictors of elder mistreatment.

**Research Design and Methods:**

Data are from a prospective study that began in the 1970s with a sample of children aged 18 months to 6 years of age. Analyses draw on data collected when child participants were in preschool and elementary school, when they were adolescents, and as adults at midlife (at ages 36 and 46, on average). Results are from bivariate correlations and multivariable path models in which variables from different life stages were entered in steps to assess their prediction of elder mistreatment.

**Results:**

All variables were significantly correlated with elder mistreatment. In a final path model, parent self-reports of physical child abuse remained a significant, unique predictor of elder mistreatment. Adolescent and adult substance use problems were also statistically significant.

**Discussion and Implications:**

Few longitudinal studies have tracked patterns of abuse over time and relationships. Findings suggest that child abuse is a risk factor for the perpetration of elder mistreatment by an adult child. Substance use problems may also increase the risk for the perpetration of elder mistreatment. Further longitudinal research is needed to replicate and extend these findings in order to advance prevention and intervention programs and policies.

**Translational Significance:** This study examined the association between child abuse and perpetration of elder mistreatment by an adult child, while also investigating measures of adolescent and adult depression and substance use problems. Results suggest that child abuse and substance abuse problems can endure as risk factors for elder mistreatment. Understanding the effects of early forms of adversity and later risk influences can guide the development of prevention and intervention programs that have potential to reduce the incidence and prevalence of elder mistreatment, a costly yet understudied public health problem.

The mistreatment of older adults, in all its forms, has a profound impact on the health and psychological well-being of victims and invariably results in losses of human rights, dignity, and, in some cases, life. With as many as 11% of community-residing older adults in the United States experiencing abuse in the past year (Acierno et al., 2010), there is a critical need for original, theory-informed research that advances knowledge of the causes, correlates, and pathways leading to the abuse and neglect of older adults (Pillemer et al., 2016).

According to reports by the Centers for Disease Control and Prevention, one in four children experience child abuse and neglect (Centers for Disease Control and Prevention, 2014), and one in five adult women experience domestic violence in their lifetimes (Breiding et al., 2015). More than 10 million women and men are impacted by family violence each year in the United States (Breiding et al., 2015). Further, a report by the National Academies estimated that between 1 and 2 million adults older than 65 years have been mistreated by a caregiver (Bonnie et al., 2003; Pillemer et al., 2016) who is often a trusted family member, such as an adult child (Jackson, 2016; Roberto, 2016, 2017). Alarming as these figures are, they underestimate the full magnitude of the problem because many acts of family violence go unreported (Acierno et al., 2010; Jackson & Hafemeister, 2015; Pillemer et al., 2016; Roberto, 2016). Still, the numbers leave no doubt that elder mistreatment is a pervasive, unrelenting, and costly public health problem that demands innovative research and comprehensive prevention and intervention programs (World Health Organization, 2002).

The tendency for abuse within families to spread across relationships is documented in numerous cross-sectional and a few longitudinal studies (T. I. Herrenkohl et al., 2020). In fact, evidence suggests that adults who were abused as children are at significantly higher risk than are others for abusing and being victimized by an intimate partner, and, possibly, for mistreating their own children (T. I. Herrenkohl et al., 2020). For some individuals, exposure to abuse is endured over many years (T. I. Herrenkohl et al., 2020; Wilkins et al., 2014). Finkelhor and colleagues (2007, 2011, 2013) coined the term “polyvictimization” to refer to patterns of abuse in which individuals are repeatedly victimized over different life stages, placing them at very high risk for serious health and mental health problems. At the same time, there are significant gaps in what is known about the repetition and spread of abuse within families, particularly across generations. An important, unanswered question is whether abuse of older adults perpetrated by adult children is more likely when those children were themselves abused. Additionally, it is unclear whether distal and proximal risk factors for elder mistreatment, such as depression and substance use problems, add to the prediction of elder mistreatment after accounting for earlier forms of abuse (McDonald & Thomas, 2013; Pillemer et al., 2016).

## Cycle of Violence Related to Elder Mistreatment

A cycle of violence within families pertains to abuse both perpetrated and experienced by parents and their adult children (Conrad et al., 2019; Dong & Wang, 2019; Korbin et al., 1995). Yet, little is known about whether the perpetration of elder abuse is indeed more common among adult children with abuse histories. What *is* known about risk and protective factors for elder abuse comes mainly from retrospective, cross-sectional studies (Easton & Kong, 2020; Pillemer et al., 2016), which are not well suited to questions about lifecourse processes because variables cannot be arranged temporally, and because retrospective data are subject to poor recall and reporting bias (Ehrensaft et al., 2003; Hardt & Rutter, 2004). In one of the very few studies to examine the relationship between child abuse and elder abuse, McDonald and Thomas (2013) found that a history of child abuse did, in fact, increase the risk for elder abuse by nearly two times, after accounting for other risk factors, including depression. Notably, child abuse was more strongly related to abuse in late adulthood than was abuse that occurred in early and middle adulthood, which suggests that early exposure to violence can be more damaging and conducive to a cycle of family violence than abuse that occurs at other life stages. The study authors concluded that a lifecourse perspective holds promise for understanding elder abuse; yet, they acknowledge that their study was limited by the use of cross-sectional data and a primary focus on elder victimization.

## Lifecourse Perspective

A central feature of the lifecourse perspective is the principle of “linked lives,” which emphasizes relational interconnectedness (Elder, 1977, 1985; Roberto & Teaster, 2017). That is, individual lives are embedded within and influenced by relationships with others (i.e., family), and shifts in dependence, independence, and interdependence can transform these relationships. For example, in early-stage families, young children are reliant upon their parents to meet most of their daily needs. As families mature, there is an increase in the bidirectional flow of instrumental, financial, and emotional support between adult children and their parents. In late-life families, parents can begin to experience declines in their physical and mental functioning and turn to their adult children for help and assistance. Assuming greater support and responsibility for parents can perpetuate ambivalent and sometimes hostile feelings about the parent–child relationship (Pillemer et al., 2019), particularly if the foundation of the relationship is compromised by past abuse. As Gordon and Brill (2001) explained, elder abuse can take the form of “reverse violence,” in which adult children who are in a position to help their parents in late life become abusive to “exact revenge” for the poor treatment they received when they were young (p. 186). Kong and Moorman (2015) suggested that unresolved trauma and weak bonds of attachment and emotional closeness between adult children and their parents can also increase the likelihood of elder mistreatment.

Substance use problems and depression are among the most common disorders for those who perpetrate abuse of older adults, as well as those who are victimized (Conrad et al., 2019; Teaster & Brossoie, 2016). Teaster and Brossoie (2016) proposed that the misuse of alcohol and other drugs can signal desperate attempts on the part of an adult child to cope with the stress and demands of caregiving, and to cover over feelings of inadequacy and unhealthy dependency on older adults. It is also important to note that substance abuse and depression are also consequences of child abuse and neglect (T. I. Herrenkohl et al., 2013, 2020), which indicates that they may play a pivotal, yet unstudied, role in the continuation and spread of abuse within families and across generations. Interestingly, a study by Kong and Moorman (2015) found that adult children who provided care to their once abusive and neglectful parents had more frequent symptoms of depression than those who had not been maltreated. This raises another potential dynamic related to stress sensitivity and the risk it poses for poor decision making and impulsive and violent behavior in a caregiving role. Unfortunately, no study has investigated these types of mediational chains that link child abuse to later elder abuse perpetration, nor has there been a systematic, longitudinal investigation of substance abuse and depression in relation to caregiving stress, and as a precursor to abuse.

## Method

### Sample

Data are from an ongoing, prospective investigation of the correlates and consequences of child maltreatment (R. C. Herrenkohl et al. 1991; T. I. Herrenkohl et al., 2013). The study began in the 1970s with a gender-balanced sample of 457 children, 18 months to 6 years of age. Subsequent assessments were completed when the children of the study were in elementary school (1980–1982), adolescence phase (1990–1992), and adulthood phase (2008–2010 and 2019–2020). Extensive data on child abuse and neglect were obtained at all waves of the study, including from parents and child welfare agencies at the start of the study in the 1970s and 1980s. The original child sample was predominantly White (80.7% White, 11.2% more than one race, and 6.8% Black/African American, Native Hawaiian, or other Pacific Islander, or unknown) and socioeconomically diverse; more than 60% of families were living below the federal poverty level upon entering the study. Data collection and analysis procedures were approved by the Institutional Review Boards at the Lehigh University, the University of Washington, and the University of Michigan.

The most recent data collection for the study was completed in 2020 when child participants had reached the age of 46 years on average. Of 303 participants retained in this most recent assessment, 52.5% were female, and 83.8% were White. Eighty percent of participants had graduated from high school or earned a Graduate Equivalency Degree, 44% had earned at least one higher education degree, and 76% were employed at least part-time. The median household income of the sample was between $60,000 and $70,000 and around a quarter of households (23%) were receiving food assistance (e.g., food stamps). Over a third of participants (36%) were receiving medical assistance because of low incomes. The analysis sample of 200 includes participants who had ongoing contact with their mothers or childhood female caregivers at the time of the 2019–2020 assessment. Around 21% of these participants were in caregiving roles (e.g., provided assistance to their older adult parents, such as working around the house, scheduling or attending appointments, and handling their financial affairs) and around 4% identified as primary caregivers for their parents.

### Measures

Prospective data on *physical child abuse* were collected from child welfare (official) records and parent self-reports. Official record reports of child maltreatment were obtained at the start of the study in the 1970s and were used in recruitment of families to the study. Just over half of all child participants (54%) were involved with child welfare agencies for abuse and/or neglect at the time of recruitment. We included a variable that identified child welfare participants (coded 1) and nonchild welfare participants (coded 0) to capture abuse reported to child welfare authorities.

Additionally, in the preschool and school-age assessments, all primary caregivers (mostly mothers) reported on the disciplining practices they and other direct caregivers used with their young children. In these two assessments, disciplining questions were anchored in different time periods. In the preschool assessment, parents were asked about their disciplining of children in (a) the past 3 months and (b) prior to the last 3 months. In the school-age assessment, parents were asked about their disciplining in the past year.

Each discipline practice was assigned a severity rating of 1 (positive) to 5 (harmful), with scores of 4 and 5 indicating the presence of abuse. Analyses for the current study combined the summed severity ratings of 12 practices from the preschool and school-age assessments. These were considered severely punishing and/or abusive (e.g., burning a child, biting a child, or hitting a child with a hard object so as to bruise). This combined measure provides one severity rating for both the preschool and school-age assessments of the study, covering time periods between birth and age 12. Scores ranged from 0 to 52, with a mean of 12.91 and an *SD* of 10.13.

Measures of *adolescent and adult depression* were derived from the Beck Depression Inventory (BDI; Beck et al., 1988). The BDI is a widely used self-report measure of depression severity for which individuals report on the extent of their feelings, including sadness (0 = I do not feel sad, 1 = I feel sad, 2 = I am sad all the time and I can’t snap out of it, 3 = I am so sad or unhappy that I can’t stand it) and disappointment (0 = I don’t feel disappointed in myself, 1 = I am disappointed with myself, 2 = I am disgusted with myself, 3 = I hate myself). Summed items total a score of 0–9 (minimal or low-level depression), 10–18 (mild depression), 19–29 (moderate depression), and 30–63 (severe depression). Scores on the adolescent measure ranged from 0 to 43, with a mean of 10.65 and an *SD* of 7.99. The scale alpha was .82. Scores on the adult measure ranged from 0 to 52, with a mean of 10.05 and an *SD* of 9.30. The scale alpha was .91.

Measures (indexes) of *adolescent and adult substance use problems* combined three items at each wave indicating whether the use of alcohol and drugs led to relationship problems and fights. In the adolescent wave of the study, participants reported on the frequency with which, in the past year, the use of drugs led to trouble with a husband/wife/boyfriend/girlfriend, trouble with friends, and getting into physical fights. Indicator variables were recoded to reflect the presence (1) or absence (0) of each problem. In the adult wave, responses indicated whether, in the past 6 months, drinking and drug use had caused problems with family and friends (yes/no), had caused problems at school or work, or led to arguments and fights. The combined adolescent measure had mean of 0.10 and an *SD* of 0.41. The adult measure had mean of 0.32 and an *SD* of 0.72.

For *elder mistreatment*, we used a modified version of the revised Conflict Tactics Scale (Beach et al., 2005; Pillemer et al., 2016; Straus et al., 1996) in which participants reported on a range of harsh and abusive practices directed at their older adult mothers in the past year (scored yes = 1 and no = 0), including verbal forms of abuse (e.g., done or said something to spite her, insulted or sworn at her), physical forms of abuse (e.g., tried to slap or hit her, threatened to hit or throw something at her), and exploitation (e.g., took or used things that belonged to her without permission). The percentage of affirmative responses ranged from a high of 12% for done or said something to spite her, insulted or sworn at her to a low of 1.5% for the two physical forms of abuse: tried to slap or hit her, and threatened to hit or throw something at her. Just 1% of participants exploited their mothers, such as taking and using their belonging, including money, bank ATM, or credit cards, without permission. Responses to these individual questions were summed to capture the overall number (variety) of abusive practices used in the past year. Scores on the composite measure ranged from 0 to 3, with a mean of 0.26 and *SD* of 0.61.

Covariates includes measures of childhood socioeconomic status (a standardized composite measure of parents’ occupational status, educational level, family income, and total rooms in the family’s home), gender (male/female), race (White/non-White), and age in adulthood.

### Analysis Procedures

Analyses consisted of bivariate correlations and a multivariable path models conducted in R (R Core Team, 2020). In the first step of the path analysis, elder mistreatment was regressed on the two measures of child abuse, as well as the covariates (childhood socioeconomic status, gender, race, age). In step 2, adolescent depression and adolescent substance use problems were added to the model with the two measures of child abuse. In step 3, adult depression and adult substance abuse were further added to the model to assess their more proximal, unique associations with elder mistreatment. Path models used maximum likelihood estimation to account for missing data.

## Results

Bivariate regressions are provided in Table 1. As shown in the table, both the official record and parent self-report measures of child maltreatment were significantly correlated with elder mistreatment (*r* = .21 and .24, respectively). Substance use problems and depression in adolescence (*r* = .28 and .16, respectively) and adulthood (*r* = .32 and .25, respectively) were also significantly correlated with elder mistreatment.

**Table 1.** Correlations of Elder Mistreatment and Other Variables

	Child abuse	Child abuse	Adolescent	Adolescent	Adulthood	Adulthood
	Official record	Parent self-report	Substance use problems	Depression	Substance use problems	Depression
Elder mistreatment	0.212**	0.240**	0.284***	0.160*	0.317***	0.245**

*Notes*: *n* = 200.

*p < .05.

**p < .01.

***p < .001.

Table 2 provides results of several path models. In the first model, both the official record measure (β = .17, *p* < .05) and the parent self-report measure of physical child abuse (β = .22, *p* < .01) predicted elder mistreatment, after controlling for childhood socioeconomic status, gender, race, and age. Variables in this model explained a significant proportion of the variance in elder mistreatment (*R*^2^ = .113, *F*(6, 193) = 4.117, *p* < .001).

**Table 2.** Results of Path Models Including Childhood, Adolescent, and Adult Variables Related to Elder Mistreatment

	First model (*R*^2^ = .113)^a^			Second model (*R*^2^ = .153)^a^			Third model (*R*^2^ = .186)^a^		
	*b*	*SE*	β	*b*	*SE*	β	*b*	*SE*	β
Covariates									
Childhood socioeconomic status	−0.015	0.016	−0.088	−0.008	0.016	−0.045	−0.008	0.016	−0.048
Gender	−0.041	0.082	−0.034	−0.054	0.080	−0.045	−0.057	0.079	−0.047
Race	−0.162	0.112	−0.100	−0.164	0.109	−0.102	−0.166	0.107	−0.103
Age	−0.036	0.023	−0.125	−0.030	0.023	−0.104	−0.028	0.022	−0.098
Child abuse									
Official record	0.210*	0.099	0.172	0.198*	0.098	0.163	0.155	0.098	0.128
Parent self-report	0.014**	0.005	0.217	0.012*	0.004	0.180	0.011*	0.004	0.173
Adolescent									
Substance use problems				0.309**	0.100	0.224	0.212*	0.105	0.153
Depression				0.003	0.006	0.037	−0.003	0.006	−0.043
Adulthood									
Substance use problems							0.159*	0.079	0.176
Depression							0.008	0.006	0.108

*Notes*: *n* = 200.

^a^*R*^2^ values shown in this table were derived from models using listwise deletion because values cannot be obtained from models using maximum likelihood estimation.

*p < .05.

**p < .01.

In the second model, which added variables for adolescent substance use problems and depression, the official record measure of child abuse (β = .16, *p* < .05) and the parent self-report measure of physical child abuse remained statistically significant (β = .18, *p* < .05). In this model, adolescent substance use problems (β = .22, *p* < .01) also predicted elder mistreatment, after accounting for other variables. Variables added to this model explained an additional 4% of variance in elder mistreatment (*R*^2^Δ = .040, *F*(8, 179) = 4.017, *p* < .001).

In the third and final model, parent self-reports of physical child abuse remained a significant predictor of elder mistreatment (β = .17, *p* < .05). Adolescent substance use problems (β = .15, *p* < .05) and adult substance use problems were also statistically significant (β = .18, *p* < .05). Variables added to this model explained an additional 3% of the variance in elder mistreatment (*R*^2^Δ = .033, *F*(10, 116) = 4.628, *p* < .001).

## Discussion

While researchers have hypothesized that abuse of children can result in higher risk for the perpetration of elder abuse (T. I. Herrenkohl et al., 2020), very few longitudinal studies have tracked patterns of abuse over time and relationships. As a result, there currently is very little empirical evidence to support or refute an intergenerational cycle of violence that includes elder abuse (Korbin et al., 1995; McDonald & Thomas, 2013; Pillemer et al., 2016; Roberto, 2016).

In the current study, we investigated associations between two measures of physical child abuse and later risk for the perpetration of mistreatment involving older adult mothers. We included measures of physical child abuse, as well as measures of adolescent and adult substance use and depression, as potential risk factors. Findings suggest that child abuse is indeed a risk factor for the perpetration of elder mistreatment and that ambivalent and sometimes hostile feeling about the parent–child relationship is a possible explanation. Obligatory family ties, even under strained circumstances, contribute to adult children’s prominence in the lives of their parents (Connidis & McMullin, 2002) and set the stage for abusive encounters (DeLiema et al., 2018). For example, rejection in everyday life, traumatic events and statements, and threat of being denied emotional or instrumental support can lead to acts of mistreatment within adult child–parent relationships and underscores the potentially long-lasting consequences of childhood abuse.

It may also be the case that adults who experienced childhood abuse choose to re-enact with their aging parents the same or similar behaviors they experienced or witnessed when they were young (Korbin et al., 1995). While the mechanisms by which childhood trauma perpetuates parental mistreatment later in life have yet to be empirically documented, shifts over time in interdependence, independence, and dependence within the parent–child dyad may contribute to feelings of ill will on the part of adult children, and thus, compromise already fragile parent–child relationships (DeLiema et al., 2018; Kong, 2018; Liu et al., 2018). More generally, findings point to the stress-induced effects of violence and related forms of trauma in childhood and the emotional impacts of child maltreatment on later perpetration of violence, including abuse directed at older adult parents.

Findings emphasize the relation between abuse and other risk factors for elder mistreatment, namely substance use problems. Substance abuse is a common disorder for those who perpetrate elder abuse (Conrad et al., 2019; Teaster & Brossoie, 2016) but is rarely investigated in the context of early-life stress associated with an individual’s exposure to child abuse. Drug and alcohol problems are likely to alter interactions and overall quality of the parent–child relationship and may exacerbate other risks, including financial dependency. For example, mistreatment may occur in late life if a parent declines or refuses to provide financial or other types of support to a vulnerable adult child with a history of addiction or mental health challenges, who then becomes increasingly desperate and lashes out at their parent physically or verbally.

### Limitations

Although this study provides new insights about abusive relationships over the life course, it has limitations, including a relatively small and homogeneous sample and a lack of racial diversity in study participants. Additionally, elder mistreatment has a relatively low base rate in this sample and mistreatment was limited to a small subset of behaviors, which were analyzed using a composite measure that prohibited analyses for different types of elder mistreatment. Further, we examined perpetration but not elder mistreatment victimization because of the design of the study and our focus on adult children caregivers. Some adult children and their aging parents may not have reached the point in their relationships when these and other forms of mistreatment will become more evident. Finally, there has been some attrition in the sample over its near 50-year time span. Loss of some participants may lessen the likelihood of detecting significant effects. Given these limitations, caution should be exercised when generalizing results beyond this sample and the geographic region it represents. Still, results advance research on the cycle of family violence and risk factors for ongoing adult relationship violence and mistreatment of older adults. Findings can help to improve the characterization of risk and protective factors for family violence that extends into late life, a developmental period that has received far less attention from researchers than other life stages. To advance research on this topic, we recommend future studies that examine the intersection of different forms of violence that occur within families, as well as factors that promote and inhibit the transmission of violence intergenerationally. Longitudinal researchers are encouraged to add elder abuse measures to ongoing studies to replicate and extend the findings of this research.
